# Impact of Specific *N*-Glycan Modifications on the Use of Plant-Produced SARS-CoV-2 Antigens in Serological Assays

**DOI:** 10.3389/fpls.2021.747500

**Published:** 2021-09-27

**Authors:** Jennifer Schwestka, Julia König-Beihammer, Yun-Ji Shin, Ulrike Vavra, Nikolaus F. Kienzl, Clemens Grünwald-Gruber, Daniel Maresch, Miriam Klausberger, Elisabeth Laurent, Maria Stadler, Gabriele Manhart, Jasmin Huber, Manuela Hofner, Klemens Vierlinger, Andreas Weinhäusel, Ines Swoboda, Christoph J. Binder, Wilhelm Gerner, Florian Grebien, Friedrich Altmann, Lukas Mach, Eva Stöger, Richard Strasser

**Affiliations:** ^1^Department of Applied Genetics and Cell Biology, Institute of Plant Biotechnology and Cell Biology, University of Natural Resources and Life Sciences, Vienna, Austria; ^2^Department of Chemistry, Institute of Biochemistry, University of Natural Resources and Life Sciences, Vienna, Austria; ^3^Department of Biotechnology, University of Natural Resources and Life Sciences, Vienna, Austria; ^4^Department of Biotechnology, University of Natural Resources and Life Sciences, Vienna, Austria; ^5^Core Facility Biomolecular & Cellular Analysis, University of Natural Resources and Life Sciences, Vienna, Austria; ^6^Institute of Immunology, University of Veterinary Medicine, Vienna, Austria; ^7^Institute for Medical Biochemistry, University of Veterinary Medicine, Vienna, Austria; ^8^Competence Unit Molecular Diagnostics, Center for Health and Bioresources, AIT Austrian Institute of Technology GmbH, Vienna, Austria; ^9^Biotechnology Section, FH Campus Wien, University of Applied Sciences, Vienna, Austria; ^10^Department of Laboratory Medicine, Medical University of Vienna, Vienna, Austria

**Keywords:** allergen, cross-reactive carbohydrate determinant, COVID-19, glycosylation, posttranslational modification, SARS-CoV-2, virus

## Abstract

The receptor binding domain (RBD) of the SARS-CoV-2 spike protein plays a key role in the virus-host cell interaction, and viral infection. The RBD is a major target for neutralizing antibodies, whilst recombinant RBD is commonly used as an antigen in serological assays. Such assays are essential tools to gain control over the pandemic and detect the extent and durability of an immune response in infected or vaccinated populations. Transient expression in plants can contribute to the fast production of viral antigens, which are required by industry in high amounts. Whilst plant-produced RBDs are glycosylated, *N*-glycan modifications in plants differ from humans. This can give rise to the formation of carbohydrate epitopes that can be recognized by anti-carbohydrate antibodies present in human sera. For the performance of serological tests using plant-produced recombinant viral antigens, such cross-reactive carbohydrate determinants (CCDs) could result in false positives. Here, we transiently expressed an RBD variant in wild-type and glycoengineered *Nicotiana benthamiana* leaves and characterized the impact of different plant-specific *N*-glycans on RBD reactivity in serological assays. While the overall performance of the different RBD glycoforms was comparable to each other and to a human cell line produced RBD, there was a higher tendency toward false positive results with sera containing allergy-related CCD-antibodies when an RBD carrying β1,2-xylose and core α1,3-fucose was used. These rare events could be further minimized by pre-incubating sera from allergic individuals with a CCD-inhibitor. Thereby, false positive signals obtained from anti-CCD antibodies, could be reduced by 90%, on average.

## Introduction

The worldwide deployment of vaccination programs against coronavirus disease 2019 (COVID-19) is one of the key measures in the fight against the current pandemic. Immunization of the population in a fast and controlled manner can protect individuals from severe COVID-19 and simultaneously limit the possibility of spreading the virus. To test the success of vaccination campaigns and monitor the immune response of vaccinated and naturally infected people, it is essential to measure the antibody titers against SARS-CoV-2 over time (Amanat et al., 2020). Serological assays using recombinant SARS-CoV-2 antigens are used to quantify the extent and durability of specific antibodies in the blood (Amanat et al., 2020; Klausberger et al., 2021). Subsequently, in order to monitor the levels of immunity in whole populations, billions of reliable antibody tests are needed.

Subunits of the SARS-CoV-2 spike protein are frequently used in serological assays to detect a specific immune response against the virus. As many neutralizing antibodies target the receptor binding domain (RBD) of the spike protein (Kreer et al., 2020; Liu et al., 2020; Pinto et al., 2020), the RBD is commonly used as an antigen in serological assays. Recombinant, soluble RBD variants have been purified from different expression systems including: mammalian cells, insect cells and plants (Diego-Martin et al., 2020; Li et al., 2020; Rattanapisit et al., 2020; Castro et al., 2021; Klausberger et al., 2021). Transient expression in plants such as *Nicotiana benthamiana* provides an attractive alternative to mammalian cell culture-based systems for cheap, fast and scalable production of diagnostic reagents and vaccine candidates (Stoger et al., 2014; Capell et al., 2020).

The monomeric SARS-CoV-2 spike protein is heavily glycosylated, featuring 22 *N*-glycosylation sites and numerous potential sites for *O*-glycosylation (Shajahan et al., 2020; Watanabe et al., 2020; Zhao et al., 2020; Sanda et al., 2021; Zhang et al., 2021). Within the RBD sequence, two *N*-glycosylation sites are typically *N*-glycosylated with complex *N*-glycans (Allen et al., 2021). While the overall role of the *N*-glycans for SARS-CoV-2 receptor binding and infection have been studied (Yang et al., 2020; Wang et al., 2021), the function of distinct *N*-glycans is less understood. Recent reports suggested that *N*-glycosylation at the two sites in the RBD is critical for binding to the cellular ACE2 receptor (Azad et al., 2021) and stabilization of the RBD in the “up” state that is required for ACE2 binding and cell entry (Sztain et al., 2021). Moreover, *N*-glycosylation of both sites is crucial for efficient expression of soluble RBD in *N. benthamiana*, indicating a role of the *N*-glycans in protein folding (Shin et al., 2021).

In comparison to mammalian cells, the *N*-glycan-processing pathway in the Golgi apparatus of plants is much simpler. Consequently, plant-produced recombinant glycoproteins carry quite homogenous complex *N*-glycans (Dicker and Strasser, 2015). Glycoengineering by knockdown of β1,2-xylosyltransferase (XT) and core α1,3-fucosyltransferase (FT) in *N. benthamiana* (ΔXT/FT) resulted in the production of recombinant glycoproteins carrying mainly the GlcNAc_2_Man_3_GlcNAc_2_ (GnGn) complex *N*-glycan (Strasser et al., 2008). As this Golgi-processed oligosaccharide structure serves as the basis for further modifications that are frequently found in humans, glycoengineering can be used to modulate the function of glycoproteins, such as immunoglobulins (Montero-Morales and Steinkellner, 2018). Wild-type plants, on the other hand, mainly produce complex and truncated *N*-glycans carrying β1,2-xylose and core α1,3-fucose residues. These sugar residues are part of carbohydrate epitopes present on plant, insect or helminth proteins which can be recognized by IgE antibodies and are considered as cross-reactive carbohydrate determinants (CCDs) (Platts-Mills et al., 2021). Approximately one fifth of patients with allergies develop IgE antibodies against such *N*-glycans (Altmann, 2007; Holzweber et al., 2013). Furthermore, production of high-affinity IgGs against these carbohydrate epitopes have also been observed (Bardor et al., 2003; Jin et al., 2006). While the clinical relevance of these epitopes is low and they do not appear to affect the safety and efficacy of recombinant biotherapeutics (Rup et al., 2017), these CCDs are a constant problem in allergy diagnosis where they are the frequent cause of false positives (Altmann, 2016; Aberer et al., 2017).

Here, we generated glycoforms of a truncated recombinant RBD variant called RBD-215 by transient expression in *N. benthamiana* and investigated their recognition by virus-specific antibodies in convalescent sera from SARS-CoV-2 exposed individuals. Our data show that all RBD glycoforms are highly suitable to detect IgG and IgM antibodies in convalescent sera and unspecific binding by pre-COVID-19 sera was generally low. However, the results obtained with a selected group of sera from allergic individuals producing anti-CCD antibodies indicated some risk of false positives caused by binding of anti-carbohydrate antibodies. In these cases, the production of recombinant viral antigens in glycoengineered ΔXT/FT plants, or the inhibition of CCDs, reduces the risk of false positives.

## Results

### RBD-215 Glycoforms With Different Types of Complex *N*-Glycans Can Be Efficiently Produced in *N. benthamiana*

**FIGURE 1:**
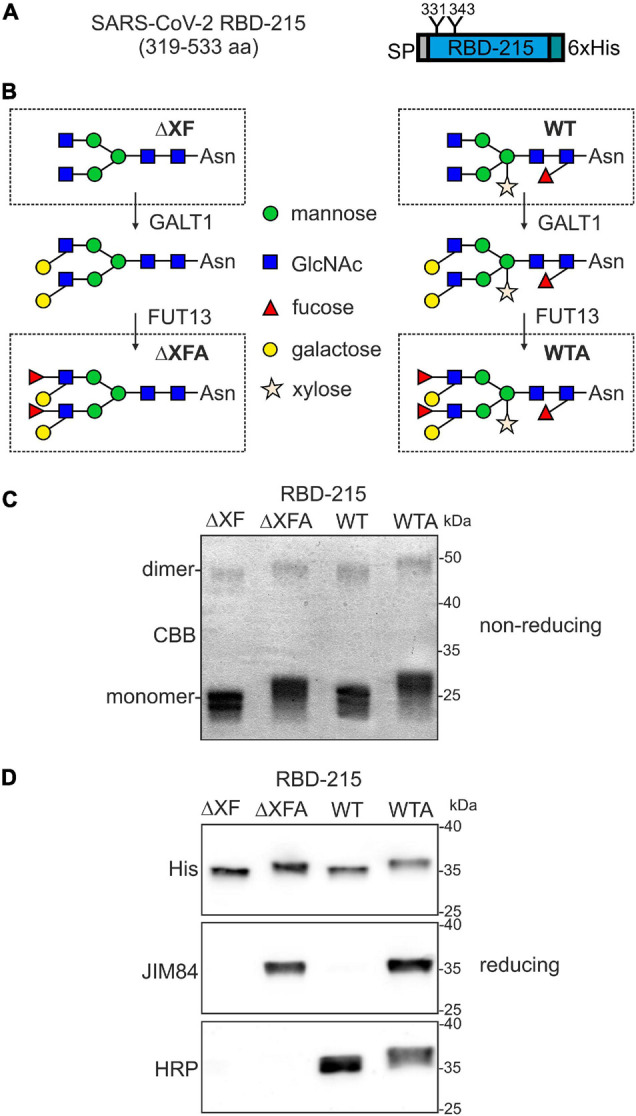
Production of RBD-215 glycoforms in *Nicotiana benthamiana*. **(A)** Schematic illustration of the expressed RBD-215 protein. The signal peptide (SP), *N*-glycosylation sites (N331 and N343) and the polyhistidine (6xHis) tag are indicated. **(B)** Cartoon illustration of the glycoengineering approach to produce the RBD-215 glycoforms. RBD-215 is expressed in wild-type (WT) or ΔXT/FT (ΔXF) plants. The Lewis A epitope is further attached by co-expression of β1,3-galactosyltransferase (GALT1) and α1,4-fucosyltransferase (FUT13). RBD-215 ΔXFA and RBD-215 WTA are RBD-215 variants carrying *N*-glycans with the Lewis A epitope. **(C)** RBD-215 glycoforms purified from the apoplastic fluid of infiltrated wild-type (WT) or ΔXT/FT (ΔXF) *N. benthamiana* leaves were subjected to SDS-PAGE under non-reducing conditions and stained with Coomassie Brilliant Blue (CBB). **(D)** Immunoblot analysis of purified RBD-215 glycoforms with anti-His (His), anti-Lewis A (JIM84) and anti-β1,2-xylose and core α1,3-fucose (HRP) antibodies.

In a previous study we observed that the most frequently used recombinant RBD variant (R319-F541) is poorly expressed in *N. benthamiana* and tends to form homodimers or aggregates (Klausberger et al., 2021). Therefore, we expressed a slightly shorter variant called RBD-215 (amino acids R319-L533). This variant lacks an unpaired cysteine at position 538 that is responsible for aberrant disulfide bridge formation (Figure 1A). This variant shows much lower amounts of homodimers/aggregates and higher yields than RBD R319-F541 upon transient expression in *N. benthamiana* leaves (Shin et al., 2021). To generate alternative RBD-215 glycoforms we infiltrated wild-type (WT) and ΔXT/FT plants, which have very low levels of β1,2-xylose and core α1,3-fucose (Strasser et al., 2008). Recombinant RBD-215 from WT (RBD-215 WT) and ΔXT/FT (RBD-215 ΔXF) are expected to differ in the presence or absence of β1,2-xylose and core α1,3-fucose residues (Figure 1B). Plant complex *N*-glycans can be further elongated by the incorporation of β1,3-galactose and α1,4-fucose, which results in the generation of Lewis A containing *N*-glycans (Strasser et al., 2007). While this complex *N*-glycan modification is only found on a small number of native plant glycoproteins (Beihammer et al., 2021), substantial amounts of Lewis A structures can be present on moss- or plant-produced recombinant glycoproteins such as human erythropoietin or β-glucocerebrosidase (Parsons et al., 2012; Castilho et al., 2013; Uthailak et al., 2021). To examine whether human sera harbor antibodies directed against Lewis A containing *N*-glycans, we generated recombinant RBD-215 modified with Lewis A structures on both *N*-glycans. The two enzymes responsible for Lewis A synthesis in *Arabidopsis*, β1,3-galactosyltransferase (GALT1) and α1,4-fucosyltransferase (FUT13) were transiently co-expressed with RBD-215 in WT and ΔXT/FT plants to generate the RBD-215 glycoforms RBD-215 WTA and RBD-215 ΔXFA (Figure 1B).

All four RBD-215 glycoforms were purified using immobilized metal affinity chromatography (IMAC) from apoplastic fluid isolated from infiltrated leaves and the overall yield was in a similar range (approximately 20 μg/g fresh weight). SDS-PAGE under non-reducing conditions revealed the presence of mainly monomeric RBD-215 with minor amounts of RBD dimers (Figure 1C). Differences in electrophoretic mobility indicated the presence of complex *N*-glycans with or without Lewis A containing *N*-glycans. The presence of Lewis A structures on RBD-215 WTA and RBD-215 ΔXFA was further confirmed using the Lewis A-specific monoclonal antibody JIM84 (Fitchette et al., 1999) (Figure 1D). RBD-215 WT did not give a signal with JIM84 showing the absence of Lewis A structures on wild-type produced RBD-215. RBD-215 WT and RBD-215 WTA reacted with antibodies against horseradish peroxidase (HRP), indicating the presence of *N*-glycans with β1,2-xylose and core α1,3-fucose residues (Strasser et al., 2004).

**FIGURE 2:**
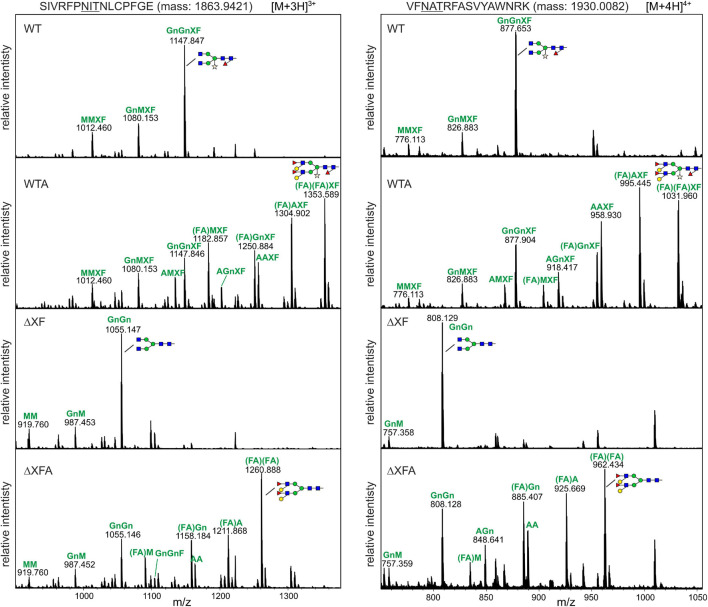
The MS spectra of the RBD-215 glycopeptides carrying the *N*-glycosylation site N331 and N343. RBD-215 glycoforms were purified by IMAC from the apoplastic fluid 3 days after infiltration, LysC and GluC digested and analyzed by MS. Major *N*-glycan peaks are illustrated with a cartoon presentation. Nomenclature of *N*-glycans is according to the ProGlycAn system (http://www.proglycan.com/). Only one *N*-glycan isoform is indicated per peak.

To analyze the *N*-glycan patterns in more detail, purified RBD-215 glycoforms were subjected to proteolytic digestion with LysC and GluC and the resulting glycopeptides SIVRFPNITNLCPFGE and VFNATRFASVYAWNRK analyzed by mass spectrometry. RBD-215 ΔXF carried mainly GnGn structures on both *N*-glycosylation sites with low amounts of truncated GlcNAc_1_Man_3_GlcNAc_2_ and Man_3_GlcNAc_2_ structures (Figure 2). On RBD-215 WT, the main structure was GnGnXF carrying β1,2-xylose and core α1,3-fucose. Co-expression of the glycosyltransferases required for Lewis A biosynthesis efficiently converted GnGn and GnGnXF to (FA)(FA) and (FA)(FA)XF, respectively. In addition, several peaks corresponding to processing intermediates or truncated complex *N*-glycans were present on RBD-215 WTA and RBD-215 ΔXFA.

### RBD-215 Glycoforms Display Comparable Functionality in ACE-2 Binding Assays

**FIGURE 3:**
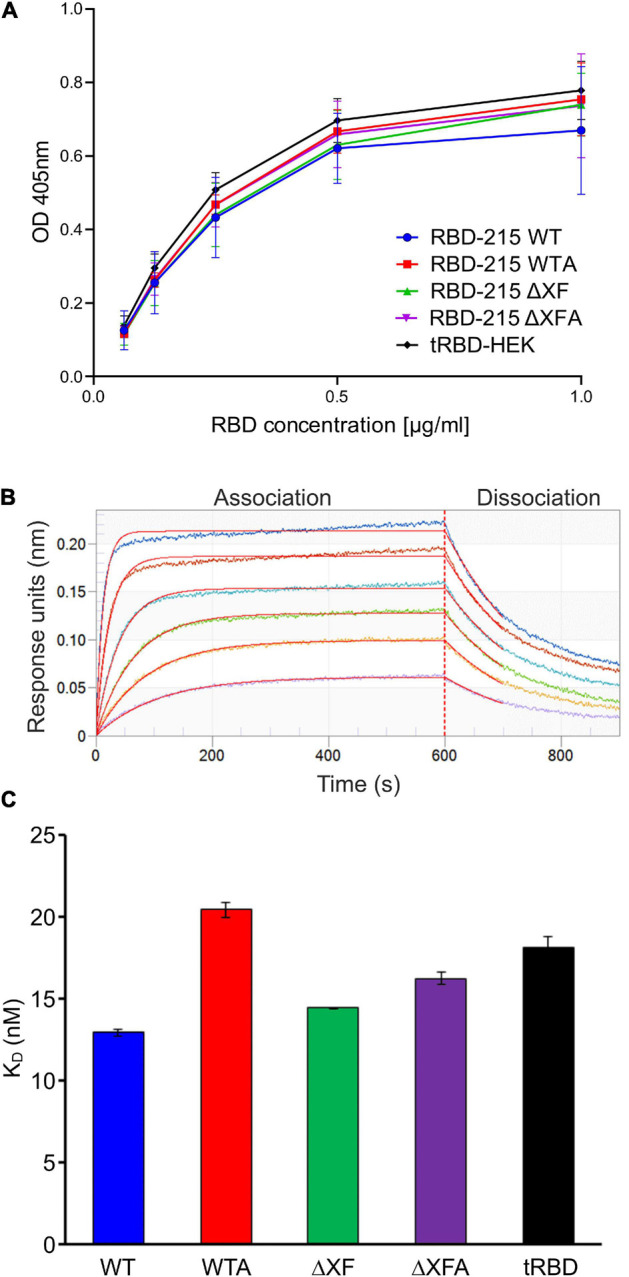
RBD-215 glycoforms display binding to ACE2-Fc. **(A)** ACE2-Fc ELISA. Binding curves of different concentrations of purified RBD-215 glycoforms and HEK293-derived tRBD (tRBD-HEK, positive control, Klausberger et al., 2021) to plates coated with ACE2-Fc. Values represent the mean ± SD (*n* = 3). **(B)** BLI analysis. Binding kinetics of the interaction between biotinylated ACE2-Fc loaded on SAX biosensors and SEC-purified monomeric RBD-215 glycoforms at a concentration range of 6.25–200 nM were determined. Representative real-time association and dissociation curves for RBD-215 ΔXF are shown. The individual curves show the association/dissociation at different concentrations of RBD-215 ΔXF. **(C)** *K*_D_ values for the interactions between ACE2-Fc and the RBD-215 glycoforms. tRBD-HEK is included for comparison. Values represent the mean ± SEM (*n* = 3).

To examine whether differences in *N*-glycan processing affect RBD binding to the SARS-CoV-2 host cell receptor angiotensin-converting enzyme 2 (ACE2) an ELISA was carried out. ACE2-Fc was coated on the ELISA plate and the binding of RBD-215 glycoforms and a HEK293-produced truncated RBD (tRBD, R319-K537) to ACE2 was measured. Overall, the binding to ACE2-Fc was comparable between all glycoforms tested and to HEK293-expressed tRBD (Figure 3A). For in-depth characterization of ACE2 binding, we used biolayer interferometry using SEC-purified monomeric variants of RBD-215 glycoforms (Figure 3B). Kinetic analysis revealed that all RBD-215 glycoforms have comparable affinities for the ACE2-Fc receptor fusion protein (13–20 nM; *n* = 3) (Figure 3C and Supplementary Table 1), which is in the same range as observed for tRBD (18.1 ± 0.6 nM; *n* = 3). Taken together, our data show that glycoengineering can be used to produce functional recombinant RBD-215 variants carrying different types of complex *N*-glycans.

### RBD-215 Glycoforms Specifically React With Convalescent Sera From COVID-19 Patients

**FIGURE 4:**
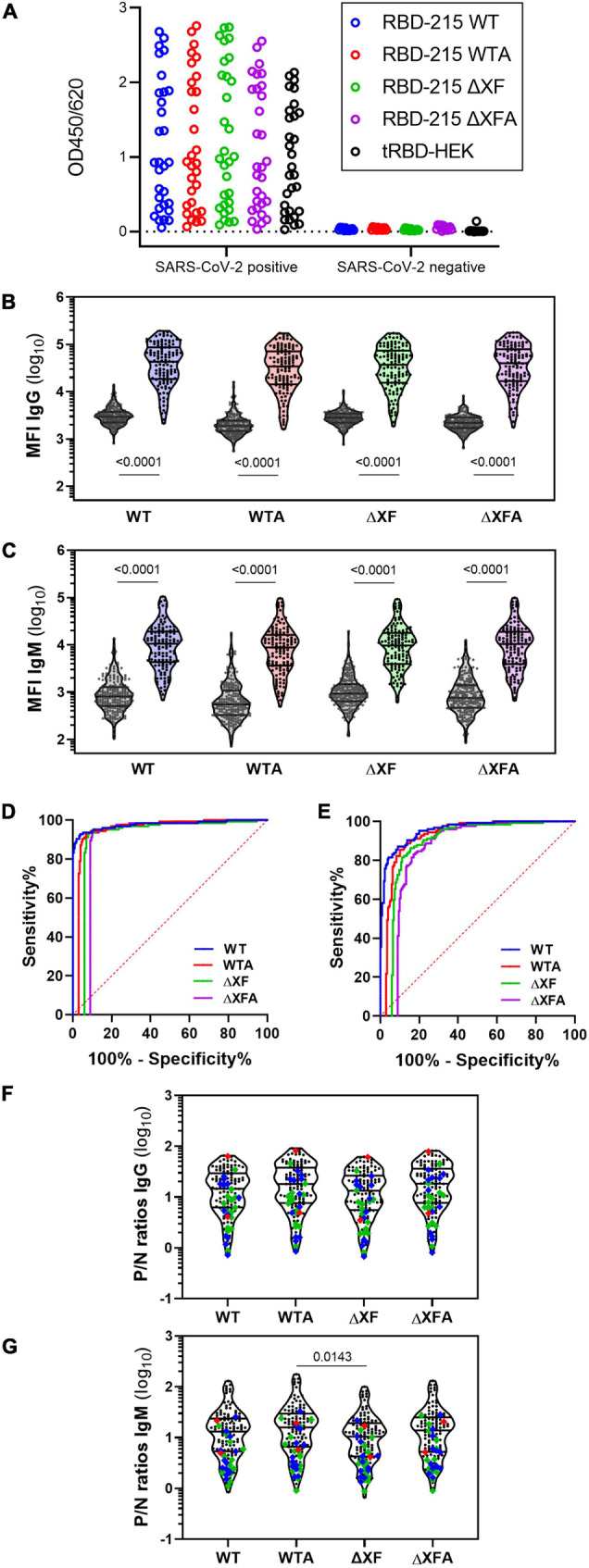
IgG and IgM antibody binding to the RBD-215 variants. **(A)** Reactivity with convalescent sera (SARS-CoV-2 positive, *n* = 30) and pre-COVID controls (SARS-CoV-2 negative, *n* = 12) with RBD-215 glycoforms and HEK293-produced tRBD. Data give the mean of three blank-corrected replicates. **(B,C)** A bead-based multiplexed seroassay using 124 convalescent sera from individuals with previous SARS-CoV-2 infection and 210 pre-pandemic sera. Violin plots give the IgG **(B)** and IgM **(C)** immunoreactivity of individual sera as median fluorescence intensity (MFI). Lines indicate the median and quartiles. A non-parametric two-tailed Mann–Whitney *U*-test was used to compare group medians of pre-pandemic and convalescent sera. **(D,E)** An overlay of the areas under the receiver-operating characteristic curve (AUC-ROC) is given for the IgG **(D)** and IgM **(E)** seroreactivity of all RBD-215 glycoforms. To ease visualization, ROC curves were horizontally nudged in respect to the ROC curve of RBD-215 WT. **(F,G)** The positive/negative (*P*/*N*) ratio of the MFI readout of each convalescent serum compared to the median of the pre-pandemic group is given for IgG and IgM. Data on the courses of disease (as per self-assessment) were color-coded (green: asymptomatic-mild, blue-moderate, red-severe; *n* = 28). Mean ranks of all groups were compared using the non-parametric Kruskal–Wallis test, followed by a Dunn’s *post hoc* test.

Next, we aimed to investigate the recognition of plant-produced RBD-215 glycoforms by SARS-CoV-2-specific antibodies in serological assays. In ELISA assays using sera from SARS-CoV-2 exposed (*n* = 30) and non-exposed (*n* = 12) individuals, we found that the RBD-215 glycoforms and HEK293-derived tRBD displayed similar IgG binding patterns (Figure 4A).

Using a bead-based multiplexed binding assay, SARS-CoV-2 specific IgG and IgM antibodies were quantified in sera from a larger cohort of SARS-CoV-2 exposed (*n* = 124) and non-exposed (*n* = 210) individuals (Figures 4B,C). The performance of the glycoengineered RBD-215 antigens in diagnostic tests was assessed through comparing receiver operating characteristic (ROC) curves and the analysis of the area under the respective ROC curve (AUC). Area under the ROC curves are performance metrics that indicate the capability of a diagnostic test to discriminate between two populations (Klausberger et al., 2021). All four RBD-215 glycoforms demonstrated AUC values of >0.98 for IgG, demonstrating their high suitability as diagnostic antigens to detect a specific immune response against SARS-CoV-2 (Figure 4D and Supplementary Table 2). Comparable AUC values for IgG were recently reported for tRBD produced in HEK293 cells using the same experimental setup (Klausberger et al., 2021). For IgM the AUC levels were slightly lower (approximately 0.95) but comparable for all four RBD-215 antigens (Figure 4E and Supplementary Table 2). The positive/negative (*P*/*N*) ratios of convalescent sera were also similar, when compared to the median of the pre-pandemic group (Figures 4F,G).

### Effect of Anti-carbohydrate Antibodies on the Antibody Reactivity to Plant-Produced RBD-215 Glycoforms in Serological Assays

**FIGURE 5:**
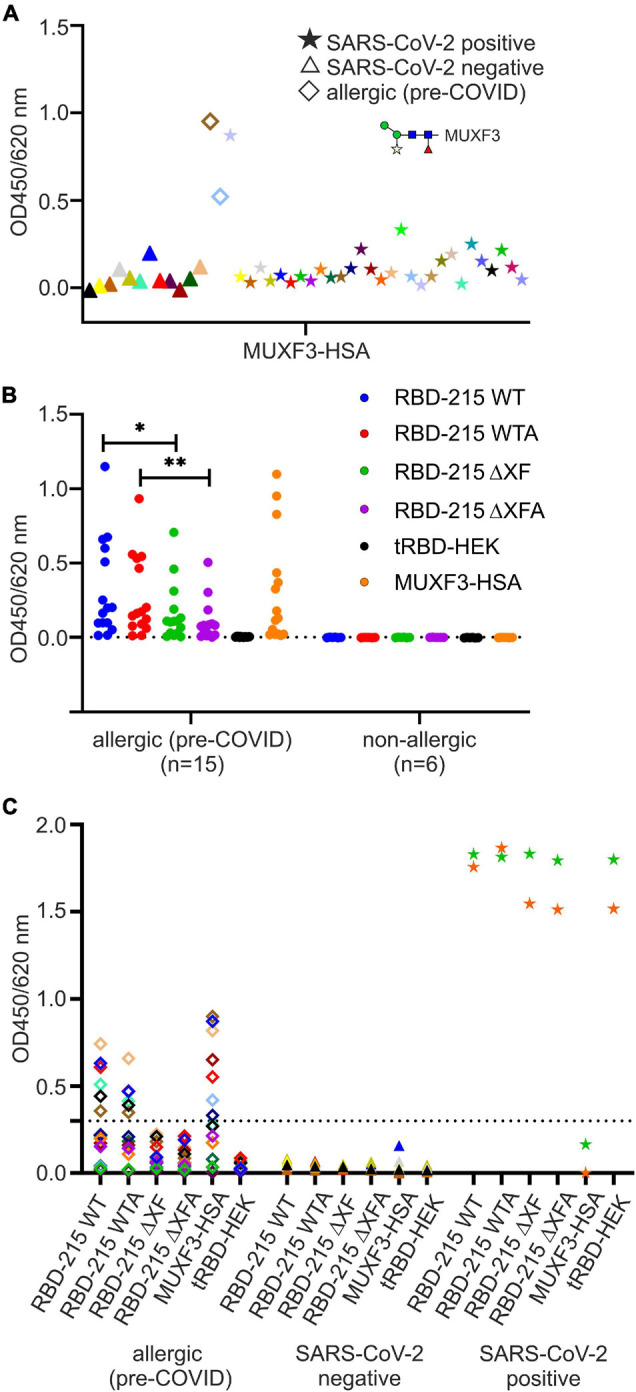
Detection of anti-carbohydrate antibodies binding to the RBD glycoforms. **(A)** Screening for anti-carbohydrate IgG antibodies in sera of SARS-CoV-2 positive (*n* = 30) and negative (*n* = 12) individuals. As antigen we used the *N*-glycan MUXF3 coupled to human serum albumin (MUXF3-HSA), because it represents a well-characterized model CCD, recognized by the vast majority of sera from CCD positive patients. Two (non-exposed) sera of allergic individuals sensitized to CCDs (allergic) served as positive controls. **(B)** Screening (IgE antibody reactivity) of a panel of sera from allergic individuals (CCD-positive, *n* = 15) to RBD-215 glycoforms, tRBD and MUXF-HSA. Sera from non-exposed non-allergic individuals (*n* = 6) served as negative controls. Data are presented as the mean of three replicates. Differences between seroreactivity to RBD-215 WT and RBD-215 ΔXF as well as for RBD-215 WTA and RBD-215 ΔXFA were tested for significance using a Student’s *t*-test (^∗^*p* < 0.05, ^∗∗^*p* < 0.01). The dashed line represents the mean from non-allergic sera plus three times the standard deviation. **(C)** IgG reactivity of allergic (*n* = 15), SARS-CoV-2 negative (*n* = 7) and of SARS-CoV-2 positive patients (*n* = 2) to the RBD-215 glycoforms, to MUXF-HSA and to tRBD. Data represent the mean of three blank-corrected replicates. The dashed line represents the mean from allergic sera to RBD-215 ΔXF/A plus three times the standard deviation.

To gain more information on the potential role of the β1,2-xylose and core α1,3-fucose and the contribution of the Lewis A structure to seroreactivity to the RBD-215 glycoforms, we analyzed a smaller cohort of sera from SARS-CoV-2 exposed (*n* = 30) and sera from non-exposed (*n* = 12) individuals for the presence of IgG antibodies to CCDs by ELISA (Figure 5A). One serum from a SARS-CoV-2 exposed individual (P1 in Supplementary Table 3) displayed high CCD reactivity to the MUXF3-HSA, in a range that was comparable to the reactivity of the control sera from the two CCD-sensitized allergic individuals. In addition, a few other sera of the SARS-CoV-2 exposed individuals displayed moderately increased reactivity indicating that CCD reactivity of CCD-sensitized individuals might contribute to the signals obtained in SARS-CoV-2 assays when using RBD-215 WT (Figure 5A and Supplementary Table 3). However, a closer look at the IgG reactivity of the MUXF3-HSA positive serum P1 to the RBD-215 glycoforms did not reveal major differences between the plant-specific RBD-215 glycoforms and tRBD produced in HEK293 cells (Supplementary Figure 1). The number of individuals with anti-CCD antibodies against β1,2-xylose and core α1,3-fucose was quite low in our cohort of SARS-CoV-2 exposed and non-exposed patients.

**FIGURE 6:**
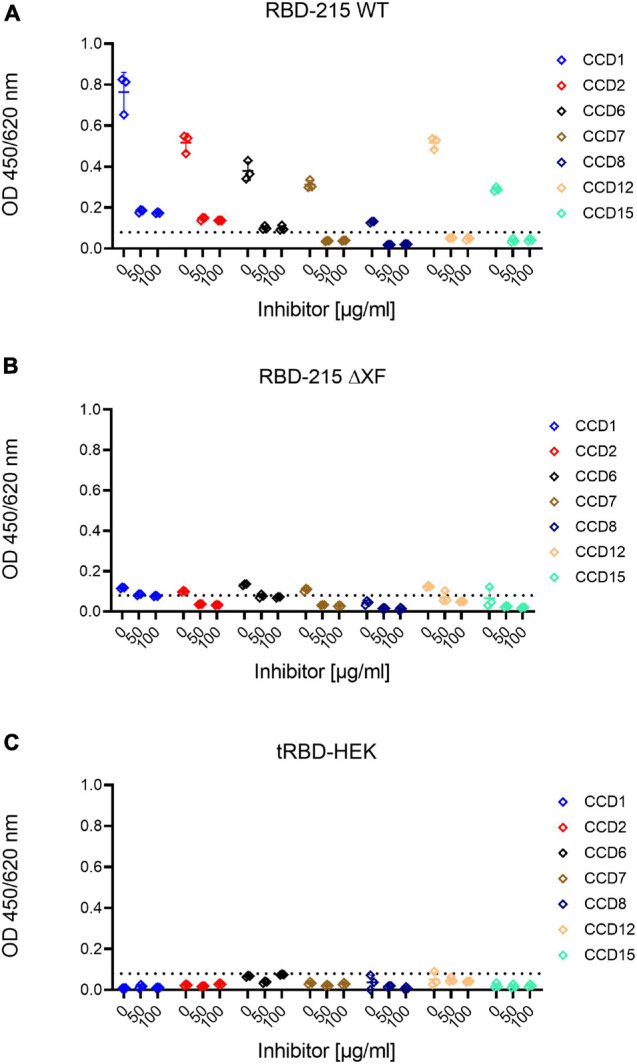
Inhibition of anti-carbohydrate IgG antibody binding by pre-incubation with MUXF3-HSA. Highly reactive sera were pre-incubated with different concentrations of MUXF3-HSA to block the binding to core α1,3-fucose and β1,2-xylose present on RBD-215 glycoforms produced in **(A)** wild-type and **(B)** ΔXT/FT plants and **(C)** HEK293 cells. The base line (dashed line) was defined as the highest signal obtained with HEK293-derived tRBD.

To assess how much of the antibody reactivity to RBD-215 glycoforms was directed against CCDs, we therefore concentrated on a selected group of sera that had been collected in the pre-COVID-19 era from fifteen allergic individuals with known CCD-reactivity. The majority of these sera displayed increased IgE levels, not only to MUXF3-HSA, but also to RBD-215 WT and RBD-215 WTA (Figure 5B and Supplementary Table 4). While the reactivity to RBD-215 ΔXF and RBD-215 ΔXFA was significantly reduced (RBD-215 WT *p* < 0.05, RBD-215 WTA *p* < 0.01), IgE binding in most sera was still higher than that obtained for tRBD produced in HEK293 cells. Next, we determined the binding of IgG antibodies present in sera from the selected allergic individuals to the RBD-215 glycoforms. Six out of fifteen individuals displayed increased IgG levels to RBD-215 WT and RBD-215 WTA, but strongly reduced reactivity to RBD-215 ΔXF and RBD-215 ΔXFA, respectively (Figure 5C and Supplementary Table 5). Despite overall reduced antibody binding capacity, the variants without plant-specific *N*-glycans showed higher antibody reactivity than the HEK293-produced tRBD. This might be due to the residual occurrence of small amounts of β1,2-xylose and core α1,3-fucose in material produced in the ΔXT/FT line, as these *N*-glycans are completely absent in HEK293 cells. The presence of the Lewis A epitope, on the other hand, had no apparent impact on antibody reactivity, suggesting that for the selected cases, it is not an immunogenic epitope. Our data show that in rare cases the presence of plant-specific modifications on viral glycoproteins could lead to false positive results in diagnostic SARS-CoV-2 antibody tests. To overcome this shortcoming, we tested the use of a CCD-inhibitor for samples which showed an increased reactivity to RBD-215 WT, but were taken in the pre-COVID era. By pre-incubating the selected sera with the model CCD, MUXF3-HSA, the IgG antibody binding capacity in all sera from allergic individuals was – on average – reduced by 90%, to a similar level as observed with HEK293 derived recombinant tRBD, which is completely devoid of β1,2-xylose and/or core α1,3-fucose residue containing *N*-glycans (Figure 6 and Supplementary Table 6).

## Discussion

A limitation of wild-type plant-produced recombinant viral antigens for serological assays is the possibility of false positives in allergic persons with antibodies against CCDs. Here, we confirm that recombinant RBD glycovariants transiently produced in *N. benthamiana* can be used as antigens in serological assays to monitor the specific antibody response against SARS-CoV-2 (Makatsa et al., 2021). Using the same set of convalescent and pre-COVID-19 sera, the antibody reactivity to *N. benthamiana* produced RBD variants was overall comparable to the antibody reactivity to RBD variants produced in other expression systems (Klausberger et al., 2021). The plant-produced RBD variants can also be used in assays with sera from individuals vaccinated with vaccines based on the SARS-CoV-2 spike glycoprotein. The different *N*-glycans attached to the plant-produced RBD variants do not alter the binding kinetics to ACE2-Fc. In fact, their affinities are comparable to tRBD produced in mammalian cells. Owing to these similar performance characteristics with ACE2-Fc, plant-produced RBD can be employed for SARS-CoV-2 surrogate virus neutralization tests (Tan et al., 2020).

Interference by carbohydrates in immunological tests is still often ignored. Yet, recombinant RBDs from different expression systems may contain carbohydrate epitopes that can be recognized by anti-carbohydrate antibodies that are commonly found in humans. Glycosylation of viral antigens depends on the host glycosylation machinery. For instance, the alpha-Gal epitope is another well-characterized carbohydrate moiety that is common in many non-human mammalian cell lines, but not present in plants (Butler and Spearman, 2014; Dicker and Strasser, 2015; Román-Carrasco et al., 2020). Furthermore, mammalian cell-based production systems, like CHO cells, can produce low levels of *N*-glycans carrying the sialic acid *N*-glycolylneuraminic acid (Neu5Gc). Neu5Gc is absent in humans due to a specific gene defect and humans have high levels of circulating anti-Neu5Gc antibodies (Yehuda and Padler-Karavani, 2020). Insect cells, on the other hand, produce recombinant glycoproteins with core α1,3-fucose that is also found in plants (Altmann, 1997). In a recent study, these specific protein modifications are thought to have caused an increased tendency toward false-positives than RBD variants derived from other non-human or human expression systems (Klausberger et al., 2021). Except for human cell lines such as HEK293, all these expression systems can potentially lead to the production of viral antigens with non-human and thus, potentially immunogenic glycan epitopes.

Therefore, the controlled modification of glycosylation pathways in expression systems, generally known as glycoengineering, is one way to circumvent given issues. Here, we show that the expression of RBD-215 in *N. benthamiana* ΔXT/FT plants, which have silenced α1,2-xylosyltransferase and core α1,3-fucosyltransferase gene expression, can reduce the seroreactivity of anti-CCD antibody bearing sera. Additionally, the use of 100 μg/ml of the described CCD blocker led to an average 90% inhibition of anti-CCD IgG antibodies in the serum of a set of selected CCD-positive patients. The inhibitor constitutes one way to circumvent given issues and can be used to avoid false positives with sera from allergic individuals sensitized to CCDs (Aberer et al., 2017). Moreover, it is now possible to produce viral antigens such as RBD in genome edited plants or plant cells that are completely devoid of β1,2-xylose and α1,3-fucose (Hanania et al., 2017; Mercx et al., 2017; Jansing et al., 2019; Herman et al., 2021). Alternatively, RBD and other glycosylated viral antigens can be manufactured in the presence of a pharmacological glycosylation inhibitor such as kifunensine which blocks α-mannose trimming from oligomannosidic *N*-glycans and thus the formation of processed complex *N*-glycans (Xiong et al., 2018; Kommineni et al., 2019; Shin et al., 2021). The retention of recombinant glycoproteins in the ER by attachment of a C-terminal HDEL/KDEL tetrapeptide is another frequently used strategy to avoid potential limitations associated with processing into complex *N*-glycans, which requires delivery of the respective glycoprotein to the Golgi apparatus (Ko et al., 2003; Petruccelli et al., 2006; Diego-Martin et al., 2020).

In contrast to *N*-glycans carrying β1,2-xylose and core α1,3-fucose, we provide an indication that *N*-glycans containing Lewis A epitopes are apparently not associated with elevated unspecific seroreactivity. Hence, plant-derived Lewis A appears not to be immunogenic suggesting that humans do not carry considerable amounts of anti-carbohydrate IgG and IgM antibodies directed against plant complex *N*-glycans with Lewis A structures. Lewis A structures are abundant on human glycolipids and are therefore not likely to be recognized as foreign, when presented on recombinant proteins (Gagneux and Varki, 1999). When produced in wild type plants overexpressing GALT1 and FUT13, the seroreactivity of CCD-positive individuals was reduced, suggesting that the Lewis A epitope masks CCD epitopes. This is in line with other studies showing altered immunoreactivity with β1,2-xylose and core α1,3-fucose when untrimmed mannoses are present on the α1,6-arm of *N*-glycans (Kaulfürst-Soboll et al., 2011) or trimming of GlcNAc residues is blocked (Liebminger et al., 2011). The reduced reactivity could be caused by the presence of decreased amounts of MMXF structures that are mainly recognized by anti-CCD antibodies (Jin et al., 2008).

In summary, the data shown here are relevant for the plant-based production of glycosylated viral antigens and demonstrate the suitability of all plant-produced glycoforms investigated in this study for SARS-CoV-2 serological assays, using 124 convalescent sera from individuals with previous SARS-CoV-2 infection and 210 pre-pandemic sera. Only when using a pre-selected cohort of sera from allergic persons with antibodies against CCDs, our data also point to a limitation of wild-type plant-produced recombinant viral antigens for serological assays. In these rare cases, the anti-CCD signals measured may be interpreted as false positive SARS-CoV-2 signals; however, this is easily reduced through the use of glycoengineered production plants and/or CCD-inhibitors. Plants therefore offer a valuable contribution to the production of recombinant viral antigens, which are needed worldwide in large amounts for serological assays to monitor the quality and longevity of the antibody response or in surrogate assays to determine titers of neutralizing antibodies (Tan et al., 2020), which represents a key indicator of protection against viruses. Furthermore, the fast transient expression of viral antigens in *N. benthamiana* allows to quickly respond to the evolution of viruses during a pandemic, for example to produce RBDs corresponding to SARS-CoV-2 variants of concerns.

## Materials and Methods

### Protein Expression and Purification

The generation of the pEAQ-*HT* expression vectors for RBD-215 was described previously (Shin et al., 2021). Syringe-mediated agroinfiltration of leaves from 5-week-old *N. benthamiana* wild-type or ΔXT/FT was used for transient expression of RBD-215 variants (Strasser et al., 2008). For the generation of the Lewis A structures, p43-GALT1 (*A. thaliana* β1,3-galactosyltransferase fused to an HA-tag) and p47-FUT13 (expression of *A. thaliana* β1,4-fucosyltransferase fused to GFP) were co-expressed by mixing of the agrobacteria prior to agroinfiltration. In both vectors, expression is under the control of the *A. thaliana ubiquitin 10* promoter.

For RBD purification, leaves were harvested 3 days after infiltration and intracellular fluid was collected by low-speed centrifugation as described in detail previously (Castilho et al., 2011). His-tagged RBD-215 was purified from collected intracellular fluid using a 5-ml HisTrap HP column (Sigma-Aldrich). Elution with imidazole, dialysis against phosphate-buffered saline solution (PBS) and concentration by ultracentrifugation was carried out as described in detail previously (Göritzer et al., 2019; Shin et al., 2021). Expression and purification of truncated RBD-His (tRBD) and ACE2-Fc in HEK293 cells has been described recently (Castilho et al., 2021; Klausberger et al., 2021).

### Immunoblot Analysis

Purified proteins were subjected to SDS-PAGE under reducing or non-reducing conditions. Samples to be analyzed under non-reducing conditions were not boiled prior to loading. Separated proteins were either stained with Coomassie Brilliant Blue (Sigma-Aldrich) or transferred to a nitrocellulose membrane (Cytiva) and detected using anti-His (Thermo Fisher Scientific), anti-HRP (Sigma-Aldrich), or JIM84 (Strasser et al., 2007) antibodies.

### Liquid Chromatography-Electrospray Ionization-Mass Spectrometry (LC-ESI-MS)

Purified RBD-215 proteins were *S*-alkylated with iodoacetamide and digested in solution with endoproteinases LysC (Roche) and GluC (Promega). Digested samples were analyzed using a maXis 4G QTOF mass spectrometer (Bruker) as described (Klausberger et al., 2021).

### ACE2-Fc Binding ELISA

ELISA was carried out according to standard protocols. Briefly, 96-well plates (Nunc MaxiSorp^TM^, Thermo Fisher) were coated overnight at 4°C with 250 ng/well of in-house produced ACE2-Fc in PBS. Plates were washed three times with PBS containing 0.1% (v/v) Tween-20 (PBS-T) using an automated plate washer. All subsequent steps were carried out at room temperature (RT). Wells were blocked for 1 h with PBS-T containing 3% (w/v) milk powder (MP-PBS-T). From an initial concentration of 1 μg/ml, a twofold serial dilution of the antigen samples was performed in blocking solution. After washing the plates three times with PBS-T, antigen samples were incubated for 2 h. Next, plates were washed three times and incubated for 2 h with mouse-anti-His-6 (1:2,000; Invitrogen) diluted in 3% (w/v) MP-PBS-T. Plates were washed again and incubated for 1 h with anti-mouse-HRP (1:2,500; Promega). After washing plates three times with PBS-T, 100 μl of 0.2 mM 2,2′-azino-bis(3-ethylbenzothiazoline-6-sulfonic acid) (ABTS; Sigma-Aldrich) in 50 mM phosphate-citrate buffer pH 5 was added and the reaction was stopped with 100 μl of 1% (w/v) SDS. The optical density (OD) of the ABTS oxidation reaction was measured at 405 nm. Background resulting from unspecific binding of detection antibodies was subtracted from the obtained values. Three technical replicates were performed. Data was analyzed using GraphPad Prism Version 9.0.0.

### Biolayer Interferometry Measurements

Interaction studies of RBD-215 with in-house produced biotinylated ACE2-Fc (Klausberger et al., 2021) was performed on an Octet RED96e system using high precision streptavidin biosensors (FortéBio). All assays were conducted in PBS supplemented with 0.05% (v/v) Tween 20 and 0.1% (w/v) BSA (PBST-BSA) at 25°C with the plate shaking at 1,000 rpm. The biosensors were equilibrated in PBST-BSA followed by dipping into a 34 nM solution of the respective biotinylated ACE2-Fc molecule. To determine dissociation constant (*K*_d_) values, titration of RBD-215 was performed to cover a broad concentration range around the respective *K*_d_ value. To record association rates, ACE2-loaded biosensors were submerged into twofold (6.25–200 nM) serial dilutions of RBD variants for 600 s. For dissociation, the biosensors were dipped into PBST-BSA for 100 s. Each experiment was performed in triplicates. Data were evaluated using the Octet data analysis software version 11.1.1.39 as previously described (Klausberger et al., 2021).

### Luminex Assays

The four RBD-215 glycoforms were separately coupled to MagPlex carboxylated polystyrene microspheres (Luminex Corporation) according to the manufacturer’s instructions, with the following minor modifications: 5 μg of each RBD-215 antigen was used for coupling per one million microspheres. Coupling was performed in a total volume of 500 μl in 96-Well Protein LoBind Deepwell plates (Eppendorf) and plates were incubated at 600 rpm on a Heidolph Titramax 1000 plate shaker (Heidolph). The coupled glycoforms were assayed in parallel with pre-COVID19 sera and sera from SARS-CoV-2 infected individuals (AIT cohorts). The source of the sera and the assay were described in detail recently (Klausberger et al., 2021).

### Measurement of IgG Antibody Responses to Receptor Binding Domains and MUXF3-HSA

ELISAs with convalescent and pre-COVID sera were carried out as described (Klausberger et al., 2021). Briefly, the respective MaxiSorp^TM^ 96-well plates were coated with 0.3 μg/well SARS-CoV-2 antigen or MUXF3-HSA (ProGlycAn) and incubated overnight at 4°C. Plates were washed 3 times with PBS-T and blocked for 1 h at RT with 3% (w/v) MP-PBS-T. Serum samples were diluted 1:200 in 1% (w/v) MP-PBS-T, 100 μl/well applied to assay plates and incubated for 2 h at RT while shaking. For inhibition of anti-CCD antibodies, 50–100 μg/ml of MUXF3-HSA was added to sera and pre-incubated for 30 min at RT. Plates were washed 4 times and incubated with anti-human IgG-HRP [Fc-specific, Sigma, 1:50000 in 1% (w/v) MP-PBS-T, 50 μl/well] for 1 h at RT while shaking. After washing for 4 times, freshly prepared substrate solution [substrate buffer (10 mM sodium acetate, pH 5 + 1:60 diluted TMB-stock solution (0.4% (w/v) tetramethylbenzidine (Fluka) in DMSO) + 1:300 diluted H_2_O_2_ (0.6% (v/v) in H_2_O))] was applied (150 μl/well) and incubated for 25 min at RT while shaking. Reactions were stopped with 25 μl/well sulfuric acid (1M, VWR) and their absorbance measured at 450 nm (reference wavelength: 620 nm) on a Tecan Sunrise reader using Magellan V 7.2 SP1 software. Data are presented as the mean of three replicates. For data analysis and visualization GraphPad Prism 9.0.0 was used.

### Detection of Anti-CCD IgE Antibodies

The source of sera from allergic individuals was described in a previous study (Román-Carrasco et al., 2020). MaxiSorp^TM^ 96-well plates were coated o/n at 4°C with 0.25 μg/well of SARS-CoV-2 antigens and MUXF3-HSA. MUXF3 is a well characterized model CCD, recognized by the vast majority of CCD positive individuals, because it contains the *N*-glycan structures known to be involved in IgE binding to CCDs: α1,3-fucose and β1,2-xylose (Holzweber et al., 2013). For blocking of the ELISA plates and for dilution of the sera and of the antibodies 0.1% HSA in PBS-T was used. Plates were blocked for 2.5 h at 37°C. After washing the plates with PBS-T, sera (diluted 1:20) were incubated o/n at 4°C while shaking. After washing of the plates, IgE binding was detected with an HRP-labeled monoclonal anti-human IgE antibody (1:5000; 2 h; SouthernBiotech, Birmingham, AL, United States). After washing the plates three times with PBS-T, freshly prepared TMB substrate solution was applied (150 μl/well) and samples were analyzed as described above. Data are presented as the mean of three replicates. For data analysis and visualization GraphPad Prism 9.0.0 was used. Student’s *t*-test was used to identify differences between RBD-215 WT and RBD-215 ΔXF, as well as between RBD-215 WTA and RBD-215 ΔXFA.

## Data Availability Statement

The raw data supporting the conclusions of this article will be made available by the authors, without undue reservation.

## Ethics Statement

The studies involving human participants were reviewed and approved by Ethics Committee Medical University of Vienna, Borschkegasse 8b/E06, 1090 Vienna; Ethics Committee City of Vienna, Thomas-Klestil-Platz 8, 1030 Vienna. Written informed consent for participation was not required for this study in accordance with the national legislation and the institutional requirements.

## Author Contributions

JS, JK-B, Y-JS, UV, NK, CG-G, DM, EL, MS, GM, JH, MH, and KV conducted the experiments. JS, MK, EL, CG-G, AW, IS, WG, FG, FA, LM, ES, and RS analyzed the results. JS, FG, FA, LM, ES, and RS supervised and designed the experiments. IS, AW, and CB provided material. JS and RS conceptualized the study and wrote the manuscript with support from MK, FG, IS, LM, and ES. All authors have made a substantial and intellectual contribution to the work and approved it for publication.

## Conflict of Interest

JH, MH, KV, and AW were employed by AIT Austrian Institute of Technology GmbH. FA who developed the CCD inhibitor is in a commercial relationship with companies who sell the inhibitor. The remaining authors declare that the research was conducted in the absence of any commercial or financial relationships that could be construed as a potential conflict of interest.

## Publisher’s Note

All claims expressed in this article are solely those of the authors and do not necessarily represent those of their affiliated organizations, or those of the publisher, the editors and the reviewers. Any product that may be evaluated in this article, or claim that may be made by its manufacturer, is not guaranteed or endorsed by the publisher.
