# Supplementary material for: Impact of Specific N-Glycan Modifications on the Use of Plant-Produced SARS-CoV-2 Antigens in Serological Assays
Source: Front Plant Sci. 2021 Sep 27;12:747500. doi: 10.3389/fpls.2021.747500 (PMC8503525; doi:10.3389/fpls.2021.747500)
Supplement: Supplementary file 1 [file Data_Sheet_1.pdf]

Supplementary Material

Impact of specific N-glycan modifications on the use of plant-produced SARS-CoV-2 antigens in serological assays

Jennifer Schwestka[1], Julia König-Beihammer[1], Yun-Ji Shin[1], Ulrike Vavra[1], Nikolaus F. Kienzl[1], Clemens Grünwald-Gruber[2], Daniel Maresch[2], Miriam Klausberger[3], Elisabeth Laurent[4,5], Maria Stadler[6], Gabriele Manhart[7], Jasmin Huber[8], Manuela Hofner[8], Klemens Vierlinger[8], Andreas Weinhäusel[8], Ines Swoboda[9], Christoph J. Binder[10], Wilhelm Gerner[6†], Florian Grebien[7], Friedrich Altmann[2], Lukas Mach[1], Eva Stöger[1], Richard Strasser[1,*]

Contents

Supplementary Figure 1. Reactivity of the serum from individual P1 to RBD-215 glycoforms.

Supplementary Table 1. K_D values for the interaction between ACE2-Fc and the RBD-215 glycoforms.

Supplementary Table 2. AUC values for IgG and IgM reactivity in the multiplex bead-based assay.

Supplementary Table 3. ELISA – binding to the MUXF3-HSA antigen.

Supplementary Table 4. ELISA – IgE reactivity to RBD antigens.

Supplementary Table 5. ELISA – IgG reactivity to RBD antigens.

Supplementary Table 6. Inhibition of anti-CCD IgGs with MUXF3-HSA.

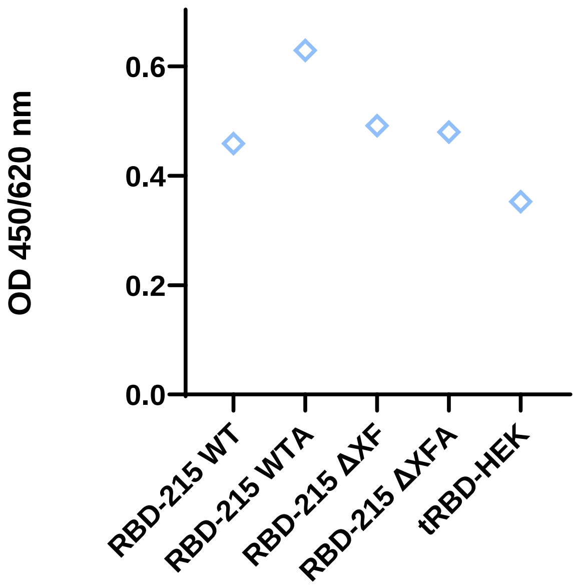

Supplementary Figure 1. Reactivity of the serum from individual P1 to RBD-215 glycoforms.

Supplementary Table 1. K_D values for the interaction between ACE2-Fc and the RBD-215 glycoforms.

	**RBD-215 WT**	**RBD-215 WTA**	**RBD-215 ΔXF**	**RBD-215 ΔXFA**	**tRBD-HEK**
K_D (nM)	12.9	20.4	14.4	16.2	18.1
SEM	0.17	0.42	0.04	0.38	0.64

Supplementary Table 2. AUC values for IgG and IgM reactivity in the multiplex bead-based assay.

IgG

**Antigen**	**AUC (*95% CI*)**<br>**Sen% (*95% CI*) at 99.5% Sp**	**p**
RBD-**215**	0.9758 *(0.9557 - 0.9960)*<br>85.5% *(78.2 – 90.6)*	<0.0001
RBD-**215WT**	0.9768 *(0.9587 – 0.9950)*<br>85.5% *(78.2 - 90.6)*	<0.0001
RBD-**215WTA**	0.9801 *(0.9655 – 0.9947)*<br>79.0% *(71.1 - 85.3)*	<0.0001
RBD-**215XFA**	0.9851 *(0.9708 – 0.9994)*<br>91.1% *(84.8 – 95.0)*	<0.0001

IgM

**Antigen**	**AUC (*95% CI*)**<br>**Sen% (*95% CI*) at 99.5% Sp**	**p**
RBD-**215**	0.9498 *(0.9267 - 0.9729)*<br>49.2% *(40.6 – 57.9)*	<0.0001
RBD-**215WT**	0.9573 *(0.9375 – 0.9770)*<br>51.6% *(42.9 - 60.2)*	<0.0001
RBD-**215WTA**	0.9558 *(0.9358 – 0.9757)*<br>49.2% *(40.6 – 57.9)*	<0.0001
RBD-**215XFA**	0.9512 *(0.9301 – 0.9722)*<br>54.0% *(45.3 – 62.6)*	<0.0001

Supplementary Table 3. ELISA binding to the MUXF3-HSA antigen. Raw data (blank-corrected) to Figure 5A.

sample	mean	± SD	sample	mean	± SD
P1	0,871	0,129	CCD7	0,953	0,035
P2	0,063	0,006	CCD13	0,521	0,036
P3	0,031	0,002	N1	-0,016	0,048
P4	0,113	0,004	N2	0,010	0,042
P5	0,039	0,006	N3	0,019	0,012
P6	0,072	0,007	N4	0,104	0,012
P7	0,028	0,019	N5	0,054	0,008
P8	0,064	0,004	N6	0,035	0,010
P9	0,038	0,025	N7	0,195	0,006
P10	0,103	0,015	N8	0,039	0,005
P11	0,057	0,005	N9	0,037	0,031
P12	0,063	0,018	N10	-0,014	0,016
P13	0,109	0,012	N11	0,051	0,007
P14	0,221	0,020	N12	0,118	0,031
P15	0,106	0,010			
P16	0,046	0,003			
P17	0,085	0,074			
P18	0,331	0,069			
P19	0,065	0,007			
P20	0,015	0,026			
P21	0,064	0,007			
P22	0,153	0,004			
P23	0,192	0,014			
P24	0,022	0,051			
P25	0,250	0,074			
P26	0,152	0,013			
P27	0,098	0,025			
P28	0,214	0,017			
P29	0,117	0,014			
P30	0,046	0,010			

Abbreviations: P, SARS-CoV-2 positive; N, SARS-CoV-2 negative; CCD, allergic individuals

Supplementary Table 4. ELISA - IgE reactivity to RBD antigens. Raw data (blank-corrected) to Figure 5B.

Antigen: RBD-215 WT				Antigen: RBD-215 ΔXF				Antigen: tRBD-HEK		
sample	mean	± SD		sample	mean	± SD		sample	mean	± SD
CCD1	0,661	0,028		CCD1	0,133	0,004		CCD1	0,0078	0,0039
CCD2	0,510	0,018		CCD2	0,707	0,044		CCD2	0,0064	0,0021
CCD3	0,253	0,010		CCD3	0,112	0,008		CCD3	0,0026	0,0017
CCD4	0,163	0,006		CCD4	0,112	0,008		CCD4	0,0029	0,0010
CCD5	0,199	0,007		CCD5	0,031	0,002		CCD5	0,0075	0,0062
CCD6	0,601	0,024		CCD6	0,313	0,026		CCD6	0,0072	0,0032
CCD7	1,151	0,054		CCD7	0,461	0,048		CCD7	0,0076	0,0023
CCD8	0,099	0,002		CCD8	0,067	0,007		CCD8	0,0025	0,0007
CCD9	0,097	0,003		CCD9	0,006	0,002		CCD9	0,0003	0,0003
CCD10	0,014	0,001		CCD10	0,014	0,000		CCD10	0,0026	0,0017
CCD11	0,053	0,004		CCD11	0,104	0,006		CCD11	0,0043	0,0041
CCD12	0,203	0,006		CCD12	0,109	0,033		CCD12	0,0055	0,0014
CCD13	0,676	0,022		CCD13	0,028	0,000		CCD13	0,0057	0,0010
CCD14	0,016	0,001		CCD14	0,008	0,001		CCD14	0,0052	0,0033
CCD15	0,099	0,008		CCD15	0,191	0,008		CCD15	0,0007	0,0003
Antigen: RBD-215 WTA				Antigen RBD-215 ΔXFA				Antigen: MUXF3-HSA		
sample	mean	± SD		sample	mean	± SD		sample	mean	± SD
CCD1	0,533	0,020		CCD1	0,083	0,002		CCD1	0,953	0,014
CCD2	0,559	0,056		CCD2	0,507	0,016		CCD2	0,328	0,091
CCD3	0,174	0,005		CCD3	0,082	0,005		CCD3	0,436	0,014
CCD4	0,146	0,006		CCD4	0,088	0,010		CCD4	0,179	0,004
CCD5	0,124	0,005		CCD5	0,019	0,001		CCD5	0,370	0,010
CCD6	0,466	0,048		CCD6	0,186	0,011		CCD6	0,829	0,012
CCD7	0,934	0,057		CCD7	0,305	0,020		CCD7	1,099	0,038
CCD8	0,090	0,005		CCD8	0,056	0,010		CCD8	0,131	0,006
CCD9	0,078	0,009		CCD9	0,003	0,001		CCD9	0,019	0,002
CCD10	0,014	0,001		CCD10	0,012	0,001		CCD10	0,023	0,001
CCD11	0,063	0,004		CCD11	0,075	0,007		CCD11	0,021	0,001
CCD12	0,202	0,007		CCD12	0,072	0,006		CCD12	0,116	0,004
CCD13	0,546	0,017		CCD13	0,017	0,003		CCD13	0,054	0,002
CCD14	0,013	0,001		CCD14	0,008	0,001		CCD14	0,014	0,001
CCD15	0,162	0,002		CCD15	0,092	0,005		CCD15	0,024	0,002

Abbreviations: CCD, allergic individuals

Supplementary Table 5. ELISA - IgG reactivity to RBD antigens. Raw data (blank-corrected) to Figure 5C.

Antigen: RBD-215 WT				Antigen: RBD-215 ΔXF				Antigen: MUXF3-HSA		
sample	mean	± SD		sample	mean	± SD		sample	mean	± SD
CCD1	0,629	0,068		CCD1	0,091	0,006		CCD1	0,871	0,176
CCD2	0,607	0,082		CCD2	0,149	0,037		CCD2	0,552	0,032
CCD3	0,027	0,018		CCD3	0,028	0,003		CCD3	0,034	0,017
CCD4	0,152	0,008		CCD4	0,062	0,002		CCD4	0,214	0,004
CCD5	0,198	0,045		CCD5	0,064	0,003		CCD5	0,174	0,149
CCD6	0,440	0,139		CCD6	0,210	0,012		CCD6	0,269	0,034
CCD7	0,357	0,120		CCD7	0,182	0,012		CCD7	0,899	0,031
CCD8	0,219	0,102		CCD8	0,059	0,022		CCD8	0,331	0,105
CCD9	0,017	0,007		CCD9	0,014	0,004		CCD9	0,004	0,002
CCD10	0,172	0,019		CCD10	0,067	0,010		CCD10	0,651	0,061
CCD11	0,213	0,116		CCD11	0,093	0,047		CCD11	0,079	0,021
CCD12	0,740	0,092		CCD12	0,223	0,025		CCD12	0,818	0,066
CCD13	0,033	0,007		CCD13	0,020	0,009		CCD13	0,418	0,021
CCD14	0,040	0,008		CCD14	0,033	0,012		CCD14	0,006	0,003
CCD15	0,509	0,108		CCD15	0,081	0,024		CCD15	0,276	0,034
N1	0,048	0,004		N1	0,037	0,005		N1	0,021	0,008
N2	0,069	0,006		N2	0,039	0,005		N2	0,027	0,012
N3	0,020	0,009		N3	0,022	0,011		N3	0,001	0,005
N4	0,039	0,002		N4	0,038	0,008		N4	0,066	0,008
N5	0,082	0,014		N5	0,051	0,011		N5	0,025	0,006
N7	0,068	0,002		N7	0,026	0,003		N7	0,158	0,006
N8	0,034	0,005		N8	0,020	0,004		N8	0,008	0,013
P28	1,830	0,158		P28	1,832	0,017		P28	0,164	0,017
P16	1,756	0,026		P16	1,546	0,061		P16	-0,001	0,004

Antigen: RBD-215 WTA				Antigen: RBD-215 ΔXFA				Antigen: tRBD-HEK		
sample	mean	± SD		sample	mean	± SD		sample	mean	± SD
CCD1	0,468	0,080		CCD1	0,191	0,021		CCD1	0,025	0,010
CCD2	0,470	0,043		CCD2	0,212	0,005		CCD2	0,086	0,019
CCD3	0,020	0,010		CCD3	0,018	0,002		CCD3	0,030	0,009

CCD4	0,143	0,015		CCD4	0,045	0,013		CCD4	0,019	0,006
CCD5	0,108	0,050		CCD5	0,055	0,054		CCD5	0,025	0,029
CCD6	0,389	0,029		CCD6	0,110	0,023		CCD6	0,058	0,007
CCD7	0,348	0,073		CCD7	0,085	0,027		CCD7	0,068	0,042
CCD8	0,208	0,089		CCD8	0,057	0,020		CCD8	0,011	0,006
CCD9	0,011	0,008		CCD9	0,007	0,003		CCD9	0,009	0,012
CCD10	0,160	0,031		CCD10	0,132	0,010		CCD10	0,027	0,006
CCD11	0,182	0,107		CCD11	0,037	0,009		CCD11	0,011	0,005
CCD12	0,658	0,086		CCD12	0,146	0,008		CCD12	0,058	0,002
CCD13	0,010	0,007		CCD13	0,015	0,008		CCD13	0,015	0,008
CCD14	0,019	0,007		CCD14	0,010	0,003		CCD14	0,010	0,003
CCD15	0,413	0,068		CCD15	0,025	0,007		CCD15	0,023	0,008
N1	0,038	0,003		N1	0,025	0,006		N1	0,016	0,003
N2	0,036	0,006		N2	0,030	0,006		N2	0,015	0,001
N3	0,016	0,008		N3	0,016	0,008		N3	0,007	0,002
N4	0,035	0,006		N4	0,030	0,005		N4	0,028	0,004
N5	0,057	0,011		N5	0,064	0,021		N5	0,046	0,006
N7	0,028	0,003		N7	0,051	0,007		N7	0,018	0,004
N8	0,068	0,037		N8	0,021	0,001		N8	0,015	0,003
P28	1,815	0,125		P28	1,794	0,060		P28	1,799	0,318
P16	1,867	0,095		P16	1,512	0,148		P16	1,517	0,267

Abbreviations: P, SARS-CoV-2 positive; N, SARS-CoV-2 negative; CCD, allergic individuals

Supplementary Table 6. Inhibition of anti-CCD IgGs with MUXF3-HSA.

Inhibition of anti-CCD IgGs with MUXF3-HSA - RBD215-WT								
		CCD1	CCD2	CCD6	CCD7	CCD8	CCD12	CCD15
**Inhibition rate [%]**	**50 µg/ml**	77,7	74,4	81,4	94,9	99,5	99,4	92,3
	**100 µg/ml**	78,4	77,9	92,0	97,1	89,7	98,6	92,0

Inhibition of anti-CCD IgGs with MUXF3-HSA - RBD215-ΔXF								
		CCD1	CCD2	CCD6	CCD7	CCD8	CCD12	CCD15
**Inhibition rate [%]**	**50 µg/ml**	33,5	77,9	59,8	88,3	100,0	71,5	85,2
	**100 µg/ml**	38,4	95,6	100,0	100,0	88,1	90,0	100,0
